# RiceChain: secure and traceable rice supply chain framework using blockchain technology

**DOI:** 10.7717/peerj-cs.801

**Published:** 2022-01-12

**Authors:** Bello Musa Yakubu, Rabia Latif, Aisha Yakubu, Majid Iqbal Khan, Auwal Ibrahim Magashi

**Affiliations:** 1 Department of Computer Science, COMSATS University Islamabad, Islamabad Campus, Islamabad, Islamabad, Pakistan; 2 College of Computer and Information Sciences, Prince Sultan University, Riyadh, Saudi Arabia; 3 Department of Rem. & General Studies, Audu Bako College of Agriculture, Danbatta, Kano, Nigeria; 4 Department of Computer Science, COMSATS University Islamabad, Islamabad, Islamabad, Pakistan; 5 Department of Crop Science, Kano University of Science and Technology, Wudil, Kano, Nigeria

**Keywords:** Agricultural supply chain, Ethereum blockchain, Food security and traceability, Rice production, Smart contract

## Abstract

The increasing number of rice product safety issues and the potential for contamination have established an enormous need for an effective strategy for the traceability of the rice supply chain. Tracing the origins of a rice product from raw materials to end customers is very complex and costly. Existing food supply chain methods (for example, rice) do not provide a scalable and cost-effective means of agricultural food supply. Besides, consumers lack the capability and resources required to check or report on the quality of agricultural goods in terms of defects or contamination. Consequently, customers are forced to decide whether to utilize or discard the goods. However, blockchain is an innovative framework capable of offering a transformative solution for the traceability of agricultural products and food supply chains. The aim of this paper is to propose a framework capable of tracking and monitoring all interactions and transactions between all stakeholders in the rice chain ecosystem through smart contracts. The model incorporates a system for customer satisfaction feedback, which enables all stakeholders to get up-to-date information on product quality, enabling them to make more informed supply chain decisions. Each transaction is documented and stored in the public ledger of the blockchain. The proposed framework provides a safe, efficient, reliable, and effective way to monitor and track rice products safety and quality especially during product purchasing. The security and performance analysis results shows that the proposed framework outperform the benchmark techniques in terms of cost-effectiveness, security and scalability with low computational overhead.

## Introduction

Public protection in agricultural products can be ensured by monitoring the production of agricultural goods and ensure the security and success of their supply chain logistics processes (Bosona & Gebresenbet, 2013). Such concerns regarding food quality and the possibility of contamination have given rise to a fresh passion for enhancing security and traceability of the agricultural supply chain. Furthermore, reliable tracking and national approval are needed for agricultural products exchanged in different countries (Opara, 2003). Critical information collection, communication, and management through the specific identification of the source and several exchanges of data through the supply chains are needed for the traceability of the agricultural supply chain (Ge, Gray & Nolan, 2015).

In the agricultural/food supply chain, it is hard to track or trace the sophistication of the data produced, packed, and forwarded by multiple intermediaries (Leng et al., 2018). Traceability as a critical policy mechanism for tracking and protecting food safety is demonstrated by food contamination and its consequences to public health (Salah et al., 2019). To improve food supply chains and its traceability in agriculture (Tian, 2016) and (Ali & Kumar, 2011) claim that the detailed collection of data by information management methods like barcodes and RFID should be used. However, the existing traceability systems for food supply chain in agriculture are mainly subject to data division and central management, which are susceptible both to changes in data and to management. If the source is contaminated and the product is quickly isolated from the supply chain, close coordination between several actors in the agricultural supply chain is needed (Wilson & Clarke, 1998).

Specific phases of food supply chains are often traceable, but data sharing between various phases is likely to be very hard and time-consuming (Ge, Gray & Nolan, 2015). As a result, many researchers have explored many other ways of ensuring easy and secure traceability of resources, these include the use of blockchain technology (Leng et al., 2018; Lin et al., 2020). However, blockchain can be described as a distributed database or ledger free from any kind of mutilation (Aggarwal et al., 2019). It is made up of blocks containing a series of transactions. Each block contains the hash of the block, some stored data, and the hash of the previous block (except for the genesis block). The hash of a block is unique and can be referred to as the signature or fingerprint of that block. Therefore, changing anything in the block will lead to a change in the entire hash (Conti et al., 2018). The hash of the previous block is used to link the blocks together. These characteristics make it very secure, since all the information entered cannot be changed easily. The nature of the information in a block depends on the type of blockchain (Fernández-Caramés & Fraga-Lamas, 2018). In this work, we will consider working with an Ethereum blockchain (Wood, 2018), as it is one of the types of blockchain that can be used both privately and publicly, unlike other types of blockchain that is public (Nakamoto, 2008).

Vitalik Buterin established the Ethereum blockchain in 2005 (Buterin, 2014). It connects private and public networks by creating accountability and enforcing private network rules. Ethereum supports smart contracts (self-triggering programs) (Atzei, Bartoletti & Cimoli, 2017). These contracts are simple programs that are stored on the blockchain and can be used to trigger an exchange of values *e.g*., coins based on certain conditions. Smart contract are programs that execute themselves when some specific conditions are made. Execution of smart contracts is associated with amount of gas consumed (Zhang et al., 2019). Gas is part of the Ethereum blockchain token that is used by the contract to pay miners for validating transactions. All Ethereum blockchain needs gas to compute and execute their smart contract and transactions. Enough gas must be paid to the miners to ensure the smooth running of the transactions, otherwise, the transactions will be terminated or will not even run (Hu et al., 2018). To avoid tampering, blocks in a blockchain must achieve agreement with one another before any modifications can be made. We have many distinct consensus algorithms, but for the sake of this study, we will focus on one of them, called Proof-of-Authority (PoA) (Xu et al., 2018).

New technical advances in the usage of blockchain technologies would offer a realistic and workable process to achieve agricultural goods traceability and remove the need for a reliable centralized controller (Fernández-Caramés & Fraga-Lamas, 2018). Blockchain technology has increased in prominence, which is why it strengthens trust between stakeholders through transparency and immutability of transactions within the supply chain and the logistics ecosystem (Waleed et al., 2021; Yakubu et al., 2021b). As it is trustworthy, secure, traceable, and tamper-proof, blockchain can be used satisfactorily in management of the agricultural and food supply chain (Leng et al., 2018; Lin et al., 2020). A large and complex network of food supply chain systems and functions comprises a broad variety of entities, including farmers, industries, processors, and consumers.

### Problem statements

While researchers have concentrated on the food and agricultural supply chain due to the troubling existence of the lengthy supply chain, it is incredibly difficult and expensive to track the origin of a commodity from raw materials to the end consumers (Bosona & Gebresenbet, 2013). The existing food supply chain approaches such as Salah et al. (2019), Wilson & Clarke (1998), and Mao et al. (2018), however, do not offer a scalable and cost-efficient avenue for agricultural food supply chain such as rice product. Therefore, a reliable and secure system needs to be established for monitoring information about the source, farming practice used and products safety over the supply chain with no assistance from third parties or central authority.

In most of the existing techniques, such as Salah et al. (2019), Tian (2016) and Mao et al. (2018), consumers lack the necessary capabilities or resources to verify or report on the quality of agricultural products in terms of defections or contaminations, forcing customers to use the product or waste it due to absence of any concise method of case reporting or consumer feedback. Consequently, developing a practical and effective method for assessing a product’s quality becomes essential in agricultural product processing and purchasing such as rice product. This will enable all stakeholders, including consumers, to actively select whom to do business within the agricultural food supply industry to maximize efficacy, a process that will be aided by economic incentive.

Other issues in the supply chain process that are not addressed in this study comprises of off chain/off-line adversary actions, revenues, and regulatory procedures for different wholesalers, processors, and retailers.

### Research objective

The overall objective of this work is to show how Ethereum blockchain technology as well as smart contracts can be used to competently monitor, control commercial transactions and business processes as well as provide avenue for consumer usability feedback seamlessly embedded into the supply chain in agriculture. An Ethereum blockchain mechanism was presented to ensure traceability and accountability in the rice supply chains. Motivated by Salah et al. (2019), Fig. 1 depicts the commodity flows in the rice supply chain, defining the stakeholders and their related position in the supply chain.

**Figure 1:**
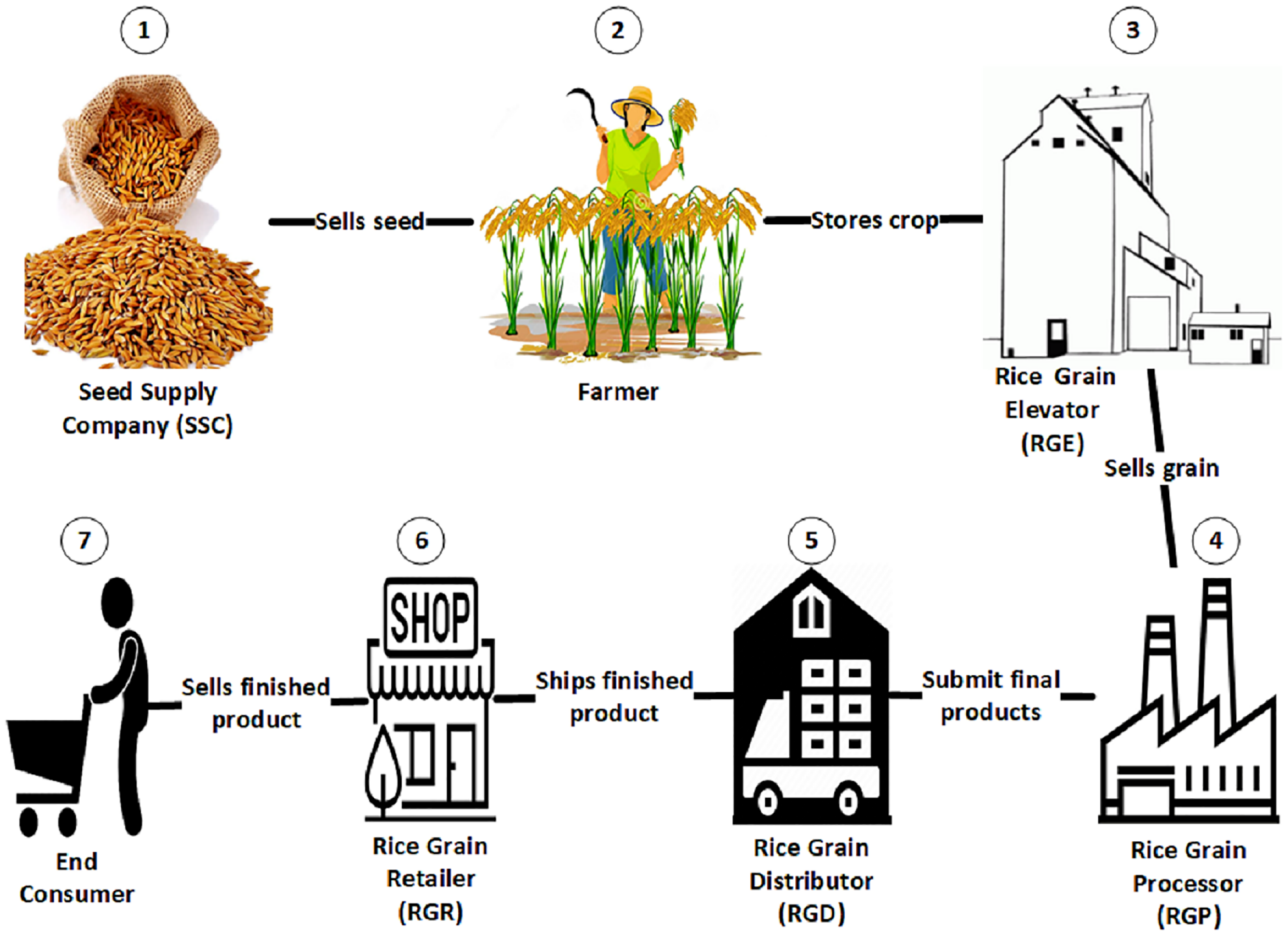
Rice grain supply chain. This diagram shows the present commodity flows in the rice supply chain, identifying stakeholders and their respective supply chain positions.

The following contributions are made in this paper:

1. With the support of Ethereum Smart Contracts, a blockchain-based traceability and transparency framework for rice supply chain is presented.
2. Given the overall framework and layout, we explore and demonstrate core facets of our blockchain approach, starring major communications among the various stakeholders.
3. A context-dependent satisfaction feedback model is presented in the rice supply chain ecosystem to calculate consumer satisfaction with a specific rice product. The model utilizes direct consumer usability experience ratings as a parameter for satisfaction assessment, which makes the model highly adaptable to dynamic situations.
4. The potentials of the smart contract algorithm were presented and evaluated to ensure proper interactions between relevant parties in the rice supply chain.

## Literature Review

Studies such as Wilson & Clarke (1998) have proposed a technological framework capable of transcending national and international boundaries. This was therefore designed to operate on a global scale, on a continuous and consistent basis, applicable to and accessible to every part of the food supply chain. In addition, studies such as Opara (2003) and Ali & Kumar (2011) proposes a framework for agricultural supply chain. It empirically analyses the role of information and communication technology (ICT) in growing farmers’ decision-making capacities in the provision of information through ICTs. In the sense of diverse farming activities in the agricultural supply chain, users of this framework have considerably stronger decision-making capabilities than non-users. However, the frameworks such as Ali & Kumar (2011), Opara (2003) and Wilson & Clarke (1998) take advantage of the continued growth in personal computer usage and the declining cost of digital communications. Similarly, the authentication mechanism used is controlled by a central administrator who is vulnerable to compromise, subjecting the data to mutilation (Curran, 2020; Praitheeshan et al., 2019).

Tian (2016) proposes a framework using RFID (Radio-Frequency IDentification) and blockchain technologies to achieve traceability and reliable information in the agri-food supply chain. The authors also examine the benefits and drawbacks of using RFID and blockchain technologies in the implementation of the agri-food supply chain traceability method. The framework effectively ensures food safety, by collecting, storing, and exchanging authentic information on agricultural food products. However, the Tian (2016) framework is primarily susceptible to system fragmentation and central administration, which are vulnerable both to data modifications and to administration (Bosona & Gebresenbet, 2013; Ge, Gray & Nolan, 2015; Lin et al., 2020).

Blockchain is a shared platform and a transparent networking model that is entirely consistent with distributed economic networks as the underlying service infrastructure (Hasan & Salah, 2018a, 2018b; Reyna et al., 2018). Besides, blockchain is a revolutionary platform that can offer a groundbreaking approach for the traceability of farm goods and commodity supply chains (Hua & Notland, 2016). Thus, the researchers in Leng et al. (2018), Mao et al. (2018) and Salah et al. (2019) introduce frameworks that uses a public and private blockchain respectively for an agricultural supply chain infrastructure. The framework in Leng et al. (2018) is based on a double-chain architecture, primarily examining the dual-chain structure and its storage mode, the resource rent-seeking and matching process, and the consensus algorithm. The frameworks demonstrate to be transparent and secure. However, the design can be cost-effective and experimentally inefficient especially in the case of double-chain architecture (Khan & Salah, 2018).

As previously mentioned, specialists have conducted a thorough examination of the supply chain and credit evaluation systems in the food industry in recent years. Not only can these systems offer consumers with the information they need, alleviate their worries, and safeguard their lives and health, but they may also be used as a decision-making tool by government agencies and other food safety authorities. Several scholars have examined the credit quality of online transactions and proposed a technique (Deng & Chen, 2017) for standardizing the operations of the online supply chain *via* the use of big data and blockchain technology (Mao et al., 2018).

According to some analyses, blockchain is the critical technology that has the greatest potential to ignite the fifth technological revolution, after steam engines, electricity, information, and Internet technology (Mao et al., 2018). Numerous studies have proposed the application of blockchain technology in the food industry as the technology matures (Zhang et al., 2020). Numerous companies and organizations are also investigating ways to “enhance” the traditional food system *via* the use of blockchain technology, a distributed, immutable record.

It was a huge deal when IBM (Edwards, 2017) announced in August 2017 that it is partnering with Nestle, Walmart (Hofmann, Strewe & Bosia, 2018; Kim, Laskowski & Nan, 2018) and other multinational food businesses to utilize blockchains in the food supply chain. A food safety collaboration was then formed in China with Fortune 500 firm JD.com and Tsinghua University, using blockchain technology to track food along the supply chain (Mao et al., 2018). To increase customer confidence in food safety throughout the food trade process, these solutions (Kim & Laskowski, 2018; Tian, 2016, 2017; Yakubu et al., 2021a) optimize business transactions and trading relationships *via* the use of robustly secure, global business networks based on blockchain technology. Nonetheless, these studies are more concerned with the traceability of traditional food supply chains (Foth, 2017; Lu & Xu, 2017) than with merchant monitoring and management in the food supply chain, as well as with customer satisfaction feedback. They are mainly concerned with ensuring the traceability and transparency of food production on a worldwide scale.

There is ample evidence in the literature that a safe and reliable mechanism is needed to trace and track agricultural goods across the agricultural supply chain as well as providing consumer usability satisfaction feedback. Regarding the monitoring and traceability of rice in the supply chain, we are introducing here a technique that employs the use of Ethereum blockchain and smart contracts and effectively carries out business activities. Our innovative approach eliminates the need for reliable, central authorities, intermediaries and offers transaction databases, and greatly improves quality and security. Table 1 provides the summary of the most related work.

**Table 1:** Summary of the related works.

Reference	Objectives	Technique used	Limitation
[4]	To provide traceability and transparency	Double-chain blockchain	Cost-effective, inefficient, double spending
[5]	To provide traceability and transparency	Blockchain	Cost-effective and inefficient
[6]	To achieve traceability and reliable information	Radio-Frequency Identification and blockchain	Susceptible to system fragmentation and central administration
[2], [7]	To analyze farmers' decision-making in supply chain	ICT gadget	Central point of failure, involvement of central controller, cost-effective and scalability issues
[8]	To provide traceability and transparency	ICT gadget	Central point of failure, involvement of central controller, and cost-effective
[23]	To provide traceability and transparency	Blockchain	Cost-effective and inefficient

## System Model

The framework is design based on private Ethereum blockchain network. Block of instructions are mined and added to the blockchain ledger by mining nodes across the private network through invoking the smart contract based on proof of authority consensus algorism. Any device that collects, validates, and executes transactions can be a mining node (Salah et al., 2019). The nodes also store the data and results of such transactions in a shared blockchain ledger that is accessible to all mining nodes. In the blockchain, smart contracts accept transactions as function calls and events so that participating stakeholders can monitor, track, and receive relevant updates where circumstances warrant. We presumed that the agricultural product data stored in the blockchain are sessional based on the expiration period of the product. Thus, data will be discarded after the expiration period completion, thereby reducing the computational overhead and enabling more on-chain transaction data. This framework aims to maintain the best working practices, correct breaches of the rice supply chain and to guarantee rice product safety to the consumer especially during product purchase. Besides, it enables the customer to give feedback on the bought goods, allowing all stakeholders engaged in the rice chain to determine if the true quality of the product is maintained throughout the supply chain or whether anything went wrong along the way. Furthermore, all data stored in the public ledger are peculiar to the rule of the associated stakeholder, thereby making the data stored none-redundant.

As depicted in Fig. 1, the framework is made up of seven (7) stakeholders which are connected to the smart contract. These includes (1) the Seed Supply Company (SSC), which is an organization that offers a wide variety of seeds specified by international standards for individual farmers. Each seed product sold by SSC is recognized using standardized identifiers such as serialized Global Trade Identification Numbers (GTIN) or similar containing the SSC prefix. (2) The farmer, he/she purchases seeds from the SSC, grows crops and deploy the smart contract with traceable uniform identification of the seeds. It is also the responsibility of the farmer to regularly monitor and record crop development information and store it in an encrypted, decentralized tamper-proof ledger such as Inter Planetary File System (IPFS) (Nizamuddin, Hasan & Salah, 2018; Salah et al., 2019). The contents of the ledger can be captured by several nodes installed on the farmland or used by the farmer (Nizamuddin, Hasan & Salah, 2018). In addition, the ledger hash is stored in the blockchain for future reference. (3) The Rice Grain Elevator (RGE), a collection and preservation center for the crop yields (rice). Apart from buying and preserving yields from the farmer, it also determines the class and quality of the crop yield. During storage of yield, the following factors are considered: temperature, humidity, and preservation time (Casino et al., 2019). (4) The Rice Grain Processor (RGP) refines the yield and measures its moisture content. RGP also removes foreign materials and transforms raw yields to finished products (Salah et al., 2019; Wilson & Clarke, 1998). (5) The Rice Grain Distributor (RGD) is an entity which purchases the final products from the RGP. It is a company that participates in the process of distributing food products to the public, to retailers (Salah et al., 2019; Wilson & Clarke, 1998). (6) The Rice Grain Retailer (RGR) purchase finished goods from the RGD in bulk, each with a Standard Traceable Identifier (STI), and sells them to the consumer in smaller quantities. Standard Identifier maintains a hierarchy which makes it easier to track goods (Salah et al., 2019; Wilson & Clarke, 1998). And (7), the end consumer an individual who purchases the packaged rice product from any available RGR and consumes it for his or her own purpose. Each rice product is accompanied with its STI and GTIN for proper traceability and identification. Each stakeholder needs to be registered with the framework and authenticated initially before it will be allowed to participate in the food chain processes. Moreover, each stakeholder participates in the blockchain network using separate Ethereum account enabling each of them to have a unique Ethereum address (EA). The stakeholders are recognized in the network by their respective EAs. Besides, EA consists of public and private keys used to digitally sign and verify the credibility of the data in each transaction and to connect each transaction to the corresponding EA or Ethereum account (Wood, 2014).

We adopt the use of traceable functionality in this work. The advantage is that it ensures that verifiable and unmodifiable information is provided to all parties concerned without the need for a central authority. By applying this across the supply chain, the total volume of rice produced sold between corresponding entities can be recorded and all transactions can be verified. For instance, there can be no change or adjustment to the amount of grain sold between entities under negotiated conditions. Furthermore, grains of multiple consistency standards cannot be combined for sale as all parties concerned are aware of their overall amount. Similarly, the day-to-day uploading *via* the file system, such as IPFS, of crops images and their land conditions, provides a digital archive that can be used to verify accepted conditions. The use of traceable identifiers per quantity of goods and the ability to track all relevant transactions between the parties concerned ensure further quality assurance monitoring.

Shipment quality can be monitored during delivery using sensor-enabled containers and packages equipped with cameras, GPS locators and 4-G connectivity. During the shipping process, the sensors will continuously relay the status of the rice products transported and provide notifications. The data notifications received are stored in the blockchain ledger where they remain immutable and accessible to all the parties involved. In addition, standard identifiers may be used to locate the exact physical position of the product. In the same way, the location of a participant can be geotagged using GPS sensors mounted within shipping containers or storage containers.

If a participant commits cheating by either transacting or recording fraudulent data, blockchain always accepts and keeps the data as it is and assigns the data to the originator of the data. Thus, if the data are later found to be fraudulent or incorrect, it is easy for all parties involved to track down the defaulting party. However, smart contracts can be used to validate the shipment or supply chain process and to place sanctions on fraudulent parties or to recommend various and proactive action to address this issue. This will provide the generation and linking of new corrective information and measures towards the fraudulent data so that to ensure accurate and unquestioned auditability.

## Secure and Traceable Rice Supply Chain Framework

**Figure 2:**
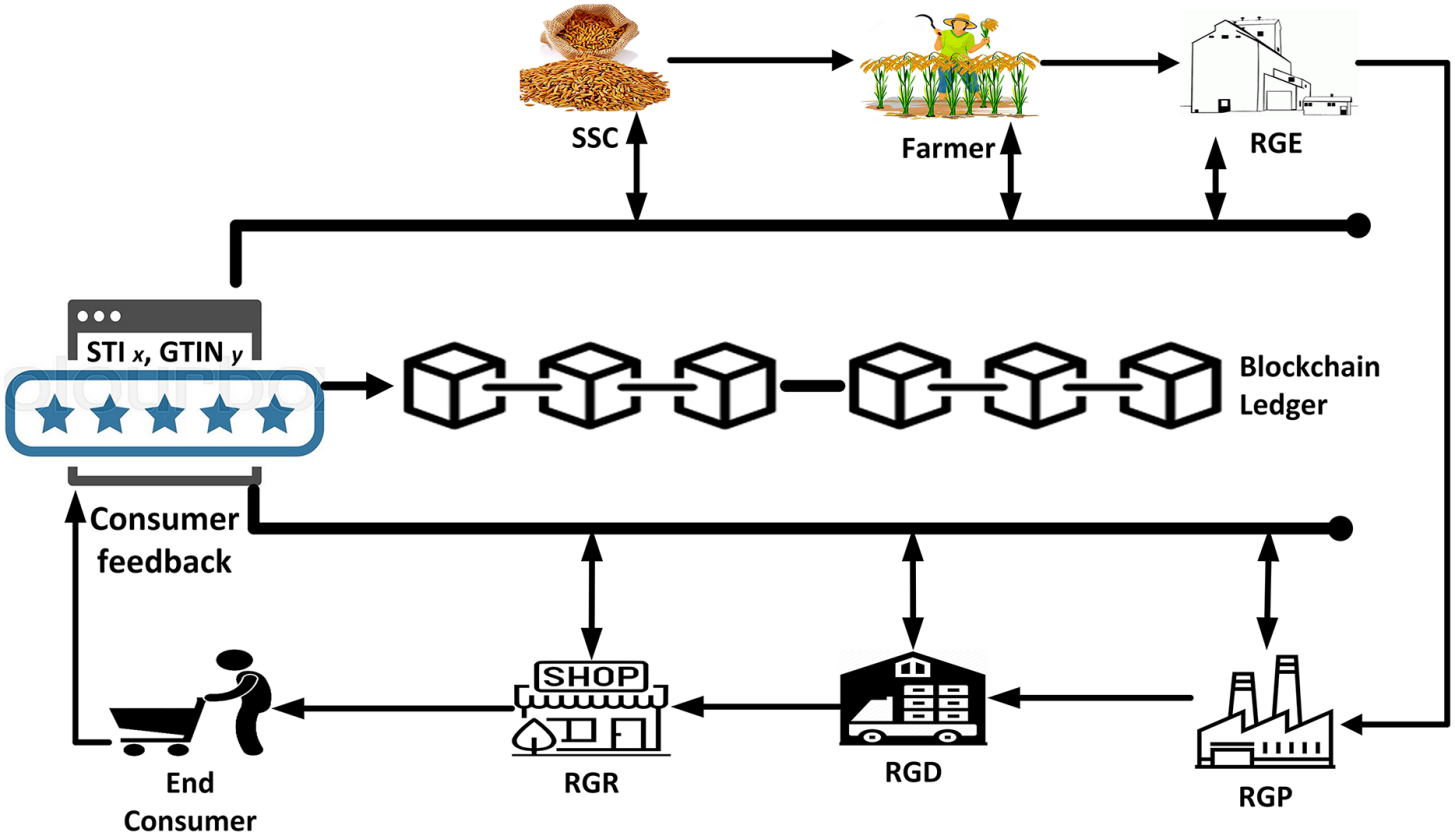
Consumer satisfaction feedback.

**Figure 3:**
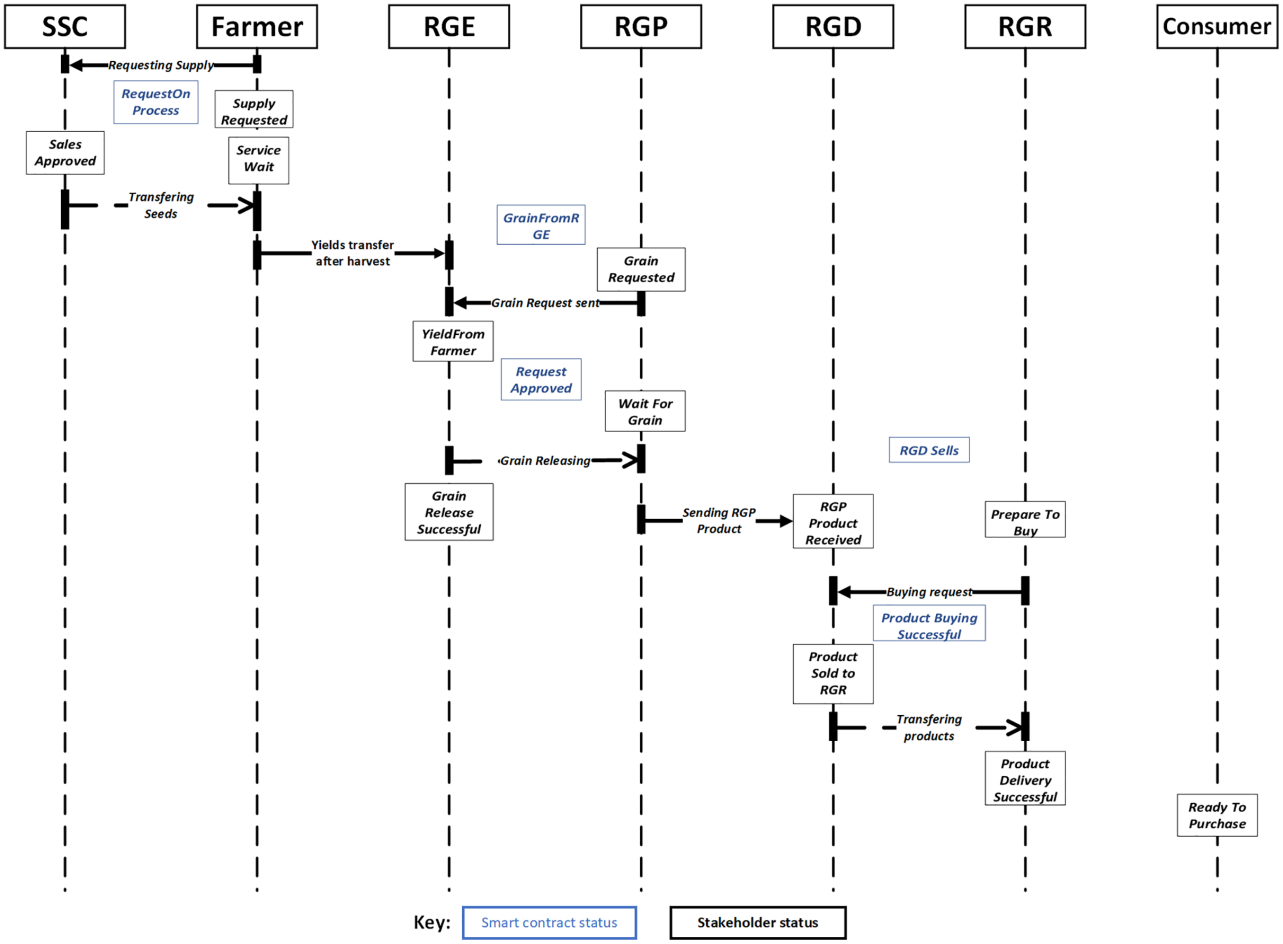
Sequence diagram of supply chain traceability process (SSC to RGR).

This section discusses the working principles of our proposed framework, followed by the consumer satisfaction feedback evaluation process as given in Fig. 2. Initially the smart contract is created and deployed, and each participating entities’ EAs are registered with the smart contract. The farmer then requests and negotiates a possible supply of rice seed with any available and appropriate SSC having this, the farmer’s status is set as $SupplyRequested$ in the smart contract as described in Algorithm 1 and Fig. 3. The smart contract verifies the authenticity of the farmer and whether payment has been made for the supply. If the farmer is confirmed to be a registered participant and a payment is made, the status of the smart contract shall be changed to $RequestOnProcess$ and the status of the farmer shall be changed to ServiceWait. And the SSC status changes to the $SalesApproved$. With this, the smart contract will notify all parties involved about the development, otherwise all status will be returned to the system’s initial position and all transactions will be revoked.

**Algorithm 1:** Buying seeds from SSC.

Input:		*Fm* is the set of all registered farmers *EA* of farmer *EA* of SSC Qantity, SType, SBrand, SPrice
1 ContractState *Created*		
2 Farmer’s Status: *SupplyRequested*		
3 SSC Status: *Read*		
4 Consider only $f \in Fm$ *i.e*. registered farmers		
5 If $farmer = registered\;and\;SPrice = paid$ *Then*		
6	Contract status $\to$ *RequestOnProcess*	
7	Farmer status $\to$ *ServiceWait*	
8	SSC status $\to$ *SalesApproved*	
9	Broadcast a notification message for the seeds sales	
10 End		
11 Else		
12	Return to initial contract state and show an error	
13 end		

After harvesting, the RGE may sell rice grain to the RGP as described in Algorithm 2 and Fig. 3; at this stage, the smart contract status is $GrainFromRGE$. This is done by considering the moisture content, bin number, purchasing and shipment date of the rice grain. The RGP status is given as $GrainRequested$ while the RGE status is $YieldFromFarmer$. From Algorithm 2, the smart contract shall verify that the applicant (requesting RGP) is a valid participant, check that the sale is accepted and that the price of the grain is paid. If these conditions are true or met, then the smart contract status changes to $RequestApproved$, the RGP status changes to $WaitForGrain$, and the RGE status changes to $GrainReleaseSuccessful$. A notification message shall be sent to all active participants concerning the selling of grain to the RGP. Then the RGP sells the final product to the RGD. However, if the conditions are false or not met, then the smart contract status changes to $RequestDenied$, the RGP status changes to $RequestNotSuccessful$, and the RGE status changes to $GrainReleaseFailed$.

**Algorithm 2:** Buying grain from RGP.

Input:		${P_{RGP}}$ is the set of all registered RGP *EA* of RGP *EA* of RGE Qantity, DatePurchased, GPrice
1 ContractState: *GrainFromRGE*		
2 RGP Status: *GrainRequested*		
3 RGE Status: *YieldFromFarmer*		
4 Consider only $RGP \in {P_{RGP}}$ *i.e*. registered RGPs		
5 If $Ricegrainsale = Accepted\;and\;GPrice = paid$ *Then*		
6	Contract status $\to$ *RequestApproved*	
7	RGP status $\to$ *WaitForGrain*	
8	RGE status $\to$ *GrainReleaseSuccessful*	
9	Broadcast a notification message for the rice grain sales	
10 End		
11 Else		
12	Contract status $\to$ *RequestDenied*	
13	RGP status $\to$ *RequestNotSuccessful*	
14	RGE status $\to$ *GrainReleaseFailed*	
15	Broadcast a notification message for the rice grain sales failure	
16 end		
17 else		
18	Return to initial contract state and show an error	
19 end		

The third stage of the framework is described in Algorithm 3 and Fig. 3. Some of the critical variables used at this stage are quantity produced, production and purchase dates. Initial smart contract status at this stage is given as $RGDSales$, while RGD and RGR status are $RGPProductReceived$ and $PreparedToBuy\;respectively$. At this stage, the smart contract guarantees that only registered RGRs can buy goods. In the same way, it verifies if the sale negotiations are approved, and the payment made. If these conditions are valid or fulfilled, the smart contract will be executed where the RGD transfers the product to the RGR. As a result, the smart contract status changes to $ProductBuyingSuccessful$, while the RGD and RGR status changes to $ProductSoldTORGR$ and $ProductDeliverySuccessful$ respectively. A notification message shall be sent to all active participants concerning the selling of rice products to the RGR. However, if the conditions are not fulfilled, the smart contract status changes to $ProductBuyinDenied$, while the RGD and RGR status changes to $ProductRequestFailed$ and $ProductDeliveryFailed$ respectively. A warning message for failure is then sent to all participants.

**Algorithm 3:** Selling rice product to RGR.

Input:		${R_{RGR}}$ is the set of all registered RGR *EA* of RGD *EA* of RGR QantitySold, DatePurchased, DateProduced, ProductPayment
1 ContractState: *RGDSales*		
2 RGD Status: *RGPProductReceived*		
3 RGR Status: *PreparedToBuy*		
4 Consider only $RGR \in {R_{RGR}}$ *i.e*. registered RGRs		
5 If $Productsale = Accepted\;and\;ProductPayment = paid$ *Then*		
6	Contract status $\to$ *ProductBuyinSuccessful*	
7	RGD status $\to$ *ProductSoldTORGR*	
8	RGR status $\to$ *ProductDeliverySuccessful*	
9	Broadcast a notification message for the rice product sales	
10 End		
11 Else		
12	Contract status $\to$ *ProductBuyinDenied*	
13	RGD status $\to$ *ProductRequestFailed*	
14	RGR status $\to$ *ProductDeliveryFailed*	
15	Broadcast a notification message for the rice product sales failure	
16 end		
17 else		
18	Return to initial contract state and show an error	
19 end		

**Figure 4:**
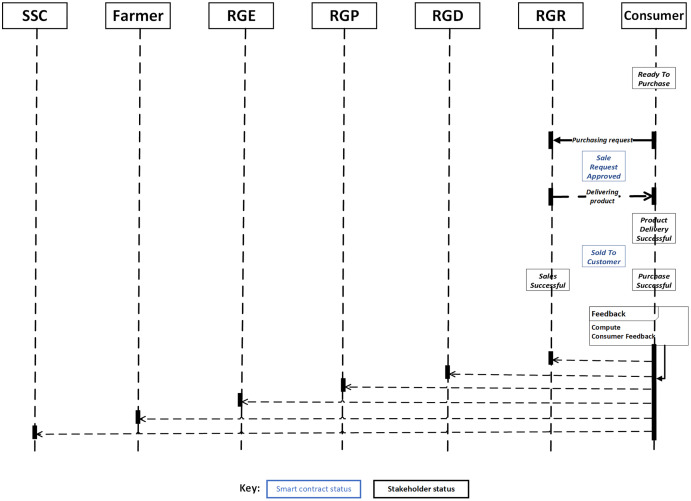
Sequence diagram of supply chain traceability process (RGR to consumer, and feedback vise-vasa).

At this stage, the final consumer who is the last participant in this system eventually purchases the final rice product from the RGR, Algorithm 4 and Fig. 4 defines the stage processes. Some of the essential criteria considered here include: Purchasing date, Sales ID, and Product ID. At this point, the initial status of the consumer is given as $ReadyToPurchase$, while that of the smart contract and RGR are $SaleRequestApproved$ and $ProductDeliverySuccessful$ respectively. Some requirements need to be met or true at this stage. This include that the smart contract guarantees that only goods from registered RGR will be made available to consumers. In addition, it shall also verify if the negotiated payment is made. If these requirements are met, the smart contract status changes to $SoldToConsumer$ while that of RGR and Consumer changes to $SalesSuccessful$ and $PurchaseSuccessful$ respectively. A notification message shall be sent to all parties involved concerning the selling of rice products to the consumer. However, if the above requirement is not successful, the smart contract status will be changed to $SaleRequestDenied$ while that of RGR and Consumer changes to $SalesFailure$ and $PurchaseFailed$ respectively. A warning message for failure will then be sent to all participants.

**Algorithm 4:** Selling rice products to consumers.

Input:		${R_{RGR}}$ is the set of all registered RGR *EA* of RGR *EA* of Consumer QantitySold, DatePurchased, SalesID and ProductID, ProductPayment
1 ContractState: *SaleRequestApproved*		
2 RGR Status: *ProductDeliverySuccessful*		
3 Consumer Status: *ReadyToPurchase*		
4 Consider only $RGR \in {R_{RGR}}$ *i.e*. registered RGRs		
5 If $RGR \in {R_{RGR}}\ and\ ProductPayment = paid$ *Then*		
6	Contract status $\to$ *SoldToConsumer*	
7	RGR status $\to$ *SalesSuccessful*	
8	Consumer status $\to$ *PurchaseSuccessful*	
9	Broadcast a notification message for the rice product sales	
10 end		
11 else		
12	Contract status $\to$ *SaleRequestDenied*	
13	RGR status $\to$ *SalesFailure*	
14	Consumer status $\to$ *PurchaseFailed*	
15	Broadcast a notification message for the rice product sales failure	
16 end		
17 else		
18	Return to initial contract state and show an error	
19 end		

### Satisfaction feedback evaluation

Consumer ${{\cal C}_i}$’s satisfaction feedback $Sf_{x,y}^i$ on a rice product $i$ bought from a given RGR with STI and GTIN of $x$ and $y$, respectively, can be assessed using direct consumer usability experience ratings, which are context-dependent as depicted in Figs. 2 and 4. The primary contexts explored in this section are how the quality of rice products is maintained in relation to functional characteristics such as uniformity of size and shape for each rice grain product. Consumers may provide comments on a rice product’s functional characteristics after direct use in this assessment. Uniformity in size and shape, whiteness, long and thin uncooked grains (*i.e*., long, and slender), and round and fat cooked grains are regarded as features of high-quality rice (*i.e*., bold cooked grains) (Custodio et al., 2019). Additional characteristics include increased satiety (*i.e*., making you feel full faster), volume expansion rather than grain elongation enhancement, and so on. Thus, ${E_{x,y}}$ represents the current ${{\cal C}_i}$ usability experience with a rice product bought from a particular RGR with STI and GTIN of x and y at a specific location, depending on the established context of functional characteristics.

Given the experience assessment scale in Table 2, the proposed model uses the existing ${{\cal C}_i}$ usability experience ${E_{x,y}}$’s value between 0 and 1. Table 2 shows all consumer usability experience values to which ${{\cal C}_i}$ can assign an ${E_{x,y}}$ value. When a given ${{\cal C}_i}$ has usability experience over a rice product $i$, it assigns a value ranging from 0.6 to 1, with 1 being a fully appreciated experience. Similarly, when identifying harmful or bad experience over a rice product $i$, then ${{\cal C}_i}$ can assign a value between 0 and 0.5, with 0 indicating an entirely disparaging experience and 0.5 reflecting a very minor degree of disparage. These values, which are allocated to ${E_{x,y}}$ are utilized to compute ${{\cal C}_i}$ satisfaction feedback $Sf_{x,y}^i$ over a rice product $i$.

**Table 2:** Experience assessment scales.

Values	Experience level
0	Entirely disparage
0.1	Absolute disparage
0.2	Severe disparage
0.3	High disparages
0.4	Relatively high disparage
0.5	Relatively low disparage
0.6	Relatively low appreciation
0.7	Relatively high appreciation
0.8	High appreciation
0.9	Strong appreciation
1	Absolute appreciation

From Eq. (1), the parameters ${a_{x,y}}$ and ${b_{x,y}}$ are adjusted based on trust decay while considering the existing consumer experience ${E_{x,y}}$.

(1) $$\matrix{ a_{x,y}=\beta^{-d\Delta t} \times a_{x,y}(old)+E_{x,y} \\ b_{x,y}=\beta^{-d\Delta t} \times b_{x,y}(old)+ (1+ E_{x,y})}$$

where *d* is the decay factor, $\Delta t$ denotes the trust update cycle and $\beta^{-d\Delta t}$ is the exponential decay on old values of ${a_{x,y}}$ and ${b_{x,y}}$, while ${E_{x,y}}$ and $1 - {E_{x,y}}$ contribute to positive and negative experiences respectively.

Hence, the satisfaction feedback $Sf_{x,y}^i$ of consumer ${{\cal C}_i}$ towards a rice product $i$ purchased from a given RGR with STI and GTIN of $x\ {\rm and}\ y$ respectively can be expressed as:

(2) $$Sf_{x,y}^i = {{{a_{x,y}}} \over {{a_{x,y}} + {b_{x,y}}}}$$

The Figs. 3 and 4 illustrate the whole supply chain traceability process, starting with the seed purchase from the SSC and ending with the end consumer’s feedback on the product. This overview is required to comprehend the proposed model more completely. Additionally, the figures depict each step of the process cycle in a sequential fashion.

## Implementation, Results, and Analysis

This section begins with the experimental setup and deployment followed by results and analysis subsections to measure the logic and performance of the proposed framework.

### Experimental setup and deployment

The experiments were designed to evaluate our proposed model using divide-and-conquer approach. The experiments were conducted with solidity language on remix IDE (Remix, 2019) using $metamask$ (Metamask, 2019) Ethereum wallet and a chrome extension that makes the interactions with Ethereum networks user-friendly (Hu et al., 2018). The remix IDE environment provides facilities for testing and debugging smart contracts before they could be deployed. Similarly, a Rinkeby (2019) $testnet$ was used to alternatively emulate the blockchain for the testing. This $testnet$ was build based on PoA consensus algorithm. With this testnet, we can have the real-world situation simulated (Hu et al., 2018). The Ethereum nodes on the $testnet$ were provided by a gateway called Infura (2019). A Truffle Framework (Truffle, 2019) was used to expedite smart contracts testing, as well as $testnet$ switching.

During testing and validation of the proposed framework, modifiers and function call in the smart contract were tested to ensure that only the desired and valid Ethereum address holder will perform the functions. Furthermore, activities in the logs are checked to ensure the proper flow of information and data.

### Results and analysis

In this section, we analyze the viability of the proposed framework in terms of security and financial expense to determine its suitability for use in a real-world environment. The section begins with the security analysis, then the cost analysis, the smart contract vulnerability analysis, future research challenges, a comparison of the proposed framework with existing works, and ends with generalization of the work and its significance.

#### Security analysis

##### Privacy

Privacy ensures that all correspondence between two parties cannot be interpreted by someone else. To ensure that only approved stakeholders have access to the digital information, contact between the various stakeholders must be kept confidential. Message encryption and decryption are accomplished in our solution through a secure SSL session following an effective handshake that guarantees the authentication of various stakeholders. The existing Public Key Infrastructure (PKI) scheme is centralized, which means it lacks transparency and relies on certificate authority for key delivery (Salah et al., 2018). The Ethereum blockchain, on the other hand, relieves us of the responsibility of using the PKI for encryption. As a result, each participating entity’s specific Ethereum Address comes with asymmetric public key pairs that are used to encrypt the exchanged messages *via* the SSL session.

##### Credibility

All messages exchanged between Ethereum network participants are tamper-proof and cannot be altered. Additionally, time stamps and separate EAs were transmitted on the chain to protect interactions between various stakeholders. As a result, the interaction is safe from both Man-In-The-Middle (MITM) and replay attacks (Hasan & Salah, 2018b).

##### Authorization

Only approved stakeholders can access all functions of the smart contract. If the call’s initiator is determined to be fraudulent, an error is generated, and all states are reset. Besides that, all subsequent interactions between the stakeholders are entirely based on an efficient handshake for authentication, resulting in a secure SSL connection.

##### Non-repudiation

All transactions and occurring events are both saved in the Ethereum public ledger, where the calls are registered and cannot be tampered by the initiator. As a result, no one can dispute their own behavior because they are all recorded in tamper-proof public ledger. Furthermore, if an attacker attempts to imitate a stakeholder’s EA, they will be caught because they do not have the correct private key to sign the message.

#### Cost analysis

A transaction expenses are incurred each time a transaction is made on the Ethereum blockchain. The remix IDE logs often contain, but are not limited to, the transaction and execution costs. Every transaction’s gas is weighed in $Gwei$ and accounted for in Ether. Transactions with a high $Gwei$ value are usually given preference by miners. The ETH gas station (Ethereum, 2021) thus offers varying purchase speeds depending on the gas price. When designing a smart contract, it is often important to measure the gas costs to avoid additional charges. Transaction costs are influenced by loops, arrays, mapping, variable storage, handling, and data types. It is critical that the solution be both feasible and effective. As a result, our solution takes advantage of blockchain’s irreversible properties and relies on events and logs instead.

Given that gas rates vary based on the day and time, it is worth noting that we are mindful of the current high gas prices because of the heavily congested network. However, compared to previous months, gas prices are relatively steady these days (May 2021). The real transaction cost is determined by the Ethereum client’s gas price. As a result, we present the expense of implementing different methods in our smart contracts in relation to the transaction and execution costs in gas as stated by the IDE. We used the ETH Gas Station’s gas prices on May 3, 2021, where the fastest, fast, average, and cheap gas prices were 44, 38, 35, and 35 $Gwei$, respectively at the time of this writing. Moreover, 1 Ether is currently worth $3180.00 on the same day. As seen in Table 3, our cost analysis presents the cost in USD based on the average gas price of 34 $Gwei$.

**Table 3:** Gas cost of Ethereum functions in USD. Displays the transaction and execution costs in Gwei The SaleRiceToConsumer function had the largest transaction and execution costs, which resulted in the highest cost of $1.32619954 in average. Even though it is the largest of the function in our solution, the cost is regarded as minimal.

Function name	Transaction gas	Execution gas	Cost in USD
*addFarmerSSCMapping*	13,593	8,776	1.159898295
*BuySeedFromSSC*	15,688	8,761	1.267772175
*getFarmerDetails*	8,282	3,465	0.609121347
*addRGP_RGEMapping*	13,593	8,776	1.159898295
*BuyGrainFromRGP*	16,026	8,741	1.28423015
*getRGPDetails*	8,278	3,460	0.608665046
*grainBuy*	7,733	3,197	0.566747508
*addRGR_RGDMapping*	13,589	8,771	1.159441993
*SaleGrainToRGR*	16,040	8,864	1.291333938
*getRGRDetails*	8,274	3,456	0.608208744
*RGRbuy*	7,729	3,193	0.566291206
*SaleRiceToConsumer*	16,423	9,153	1.32619954
*getPurchaseDetails*	7,845	3,028	0.563802288
*ConsumerBuy*	8,245	3,709	0.619802956

Table 3 displays the transaction and execution costs in $Gwei$. The $SaleRiceToConsumer$ function had the largest transaction and execution costs, which resulted in the highest cost of $1.32619954 in average. Even though it is the largest of the function in our solution, the cost is regarded as minimal. The $SaleRiceToConsumer$ function cost a lot of gas due to the generation of a random sales and product IDs dynamically. Thus, apart from the $SaleRiceToConsumer$ function, the costs of all functions are less than $1.3 each for execution which is minimal if compared to the risen prices of Ethereum currencies nowadays.

#### Smart contract vulnerability analysis

The proposed Framework’s Smart Contracts were analyzed using the Oyente Security Analysis Tool (Luu et al., 2016), and the results are provided in Table 4. The tool analyses the EVM byte codes and generates an appropriate smart contract call map. The Smart Contract has been found to be free of security flaws. There was no unchecked exception to smart contracts that could lead to underflow or overflow of integer transactions. All tests have been made to ensure that gas is available throughout execution in to prevent re-entrance attacks. The analysis showed that there was no flaw in the framework that could result in timestamp dependency, transaction dependency and parity multisig bug.

**Table 4:** Vulnerability analysis report. The analysis showed that there was no flaw in the framework that could result in timestamp dependency, transaction dependency and parity multisig bug.

PARAMETERS	RESULTS
EVM Code Coverage	62%
Integer Underflow	False
Integer Overflow	False
Parity Multisig Bug 2	False
Call stack Depth Attack Vulnerability	False
Transaction Ordering Dependence	False
Time Stamp Dependency	False
Reentrancy Vulnerability	False

#### A comparison of the proposed framework with existing agricultural food supply chain solutions

Table 5 depicts the comparison analysis. The table compares the framework proposed in this article to current related techniques Salah et al. (2019) and Mao et al. (2018), in the literature. The current techniques try to address agricultural food supply traceability issues such as data integrity, information accessibility, traceability, and transparency by proposing smart contract solutions and consensus mechanisms. The techniques also discussed how their approach could be deployed in the real world with few or no technical requirements. For example, Salah et al. (2019) examine the practicality of using Ethereum-based smart contracts in conjunction with IPFS to store data while also utilizing numerous smart contracts to carry out supply chain actions, resulting in an increase in the computational complexity of their approaches. While (Mao et al., 2018) gives traders credit results that will be utilized as references for regulators’ oversight and management in the food supply chain. The approach (Mao et al., 2018) collects credit assessment text from traders *via* blockchain smart contracts and then analyzes the text directly using a deep learning network called Long Short-Term Memory (LSTM). However, Salah et al. (2019) made no mention of the proposed solution’s implementation or testing. And both Salah et al. (2019) and Mao et al. (2018) did not provide any type of study, such as cost, security analysis or Vulnerability analysis, to show that their approach is possibly viable. Similarly, consumer usability satisfaction feedback was also not considered in these techniques. On the contrary, our proposed solution (RiceChain) is not only providing effective, secure, and traceable food grain (rice product) supply chain, but it also proposed an approach for providing consumer usability feedback to enable other stakeholders to have update and make decision on each product. Besides, the model was also developed and tested utilizing Remix IDE, as well as the necessary analyses such as cost, security analysis and vulnerability analysis was carried out to demonstrate the viability of the approach.

**Table 5:** A comparison of the proposed framework with existing works. Our proposed solution is not only providing effective, secure, and traceable food grain (rice product) supply chain, but it was also developed and tested utilizing Remix IDE, as well as the necessary analyses such as cost, security analysis and vulnerability analysis was carried out to demonstrate the viability of the approach.

Features	Salah et al. (2019)	Mao et al. (2018)	RiceChain
Satisfaction feedback	No	No	Yes
Cost Analysis	No	No	Yes
Implementation and Testing	No	Yes	Yes
Security analysis	No	No	Yes
Vulnerability analysis	No	No	Yes
Low computational cost	No	No	Yes
Proof of Concept	Yes	No	Yes

**Figure 5:**
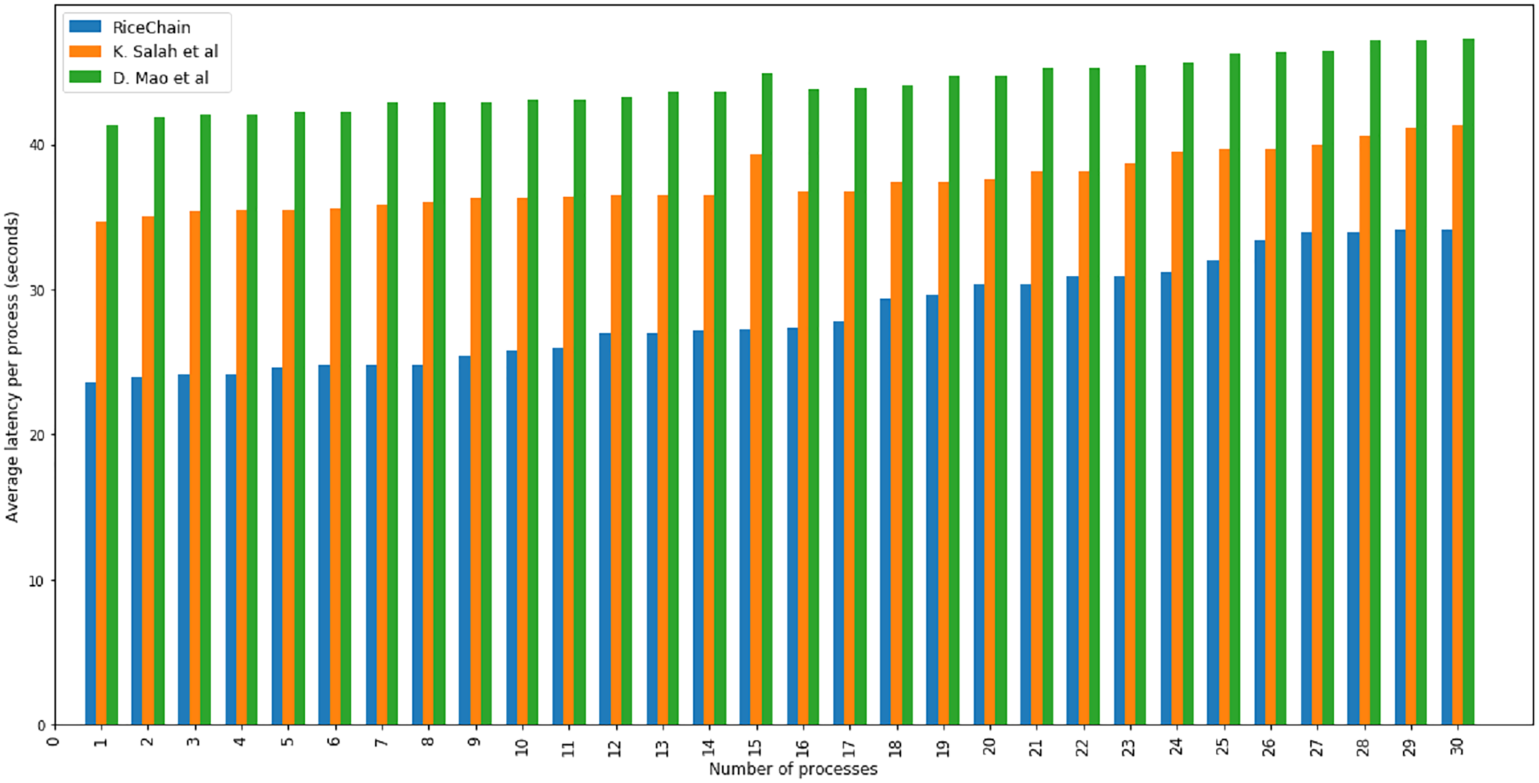
Performance analysis based on the latency of process executions.

The RiceChain model’s logic was also validated by executing number of processes in the system. The experiment begins with one complete process in the system (starting from seed procurement from SSC and ending with end customer purchase and feedback). Gradually, more process were added until we have maximum thirty (30) distinct full traceability processes in the system. The average latency of each process’s execution was determined; this includes the latencies associated with the execution of each process’s related functions. The performance of the RiceChain model was compared to that of Salah et al. (2019) and Mao et al. (2018), and the findings indicate that the RiceChain model beats the other two models in terms of the average latency of all processes executed in the tests. The findings of the performance analysis based on the latency of process executions are shown in Fig. 5.

## Discussions

To assess the feasibility of using blockchain technology in the rice supply chain, we provide a working prototype of a blockchain-based system, together with security and cost assessments. The proposed framework was developed and tested on a private Ethereum blockchain platform to meet the high visibility, transparency, and security requirements of users in the supply chain and rice grain sector. Due to the encryption of data and transactions on private blockchain systems, trust is reinforced, making it more attractive for business entities to retain their transactions. The offered solution may be customized to suit the growing expectations and requirements of different companies. To ensure high transaction and execution speeds, as well as privacy, transparency, and security, the provided smart contracts are readily modifiable and deployable on private blockchain systems. Additionally, our methodology may be used in conjunction with external oracles and proxy encryption techniques to address privacy issues.

The proposed model is capable of tracing and tracking the food supply chain connected with rice grain production in an effective manner. Additionally, it helps in the monitoring of rice product delivery and ensures that all parties involved adhere to supply chain regulations. Sensors are used to monitor sealed rice grains and other goods throughout shipping to ensure they are not contaminated. Throughout the supply chain process, all parties responsible for improper conduct are recognized, reducing the risk of food contamination.

Numerous other grain crops in the same category exhibit similar features to rice cultivation (*e.g*., Wheat, Beans, Millet *etc*.). Due to the efficiency with which the proposed model followed and monitored the rice production food supply chain, this study is also applicable to other grain food supply chains. For instance, the government might use this study to monitor and track wheat, beans, millet, and other grains supply. Thus, this study may be utilized to monitor and trace the supply chain of any grain product, not only rice grain. Additional beneficiaries of this endeavor include grain processing companies, farmers, agronomists, agricultural agencies, merchants, agricultural food authorities, and crop scientists and specialists.

Solidity was used to create smart contracts on the Ethereum blockchain platform. Due to the general nature of our method, it can be readily adapted for use on a variety of blockchain systems with little effort. Given the effectiveness of our proposed model in limiting rice contamination to a minimum, agricultural authorities may seek to enhance this achievement by adopting additional rules aimed at further reducing grain food contamination (*e.g*., rice).

## Conclusion

In this article, we provided a fully distributed framework for a secure and traceable rice supply chain. The framework uses Ethereum blockchain and smart contracts for monitoring, tracking and implementation of commercial transactions to eradicate intermediaries. Architectural design and development algorithm were presented and implemented to show the processes flow of the traceable rice supply chain. Similarly, the model includes a concept for consumer satisfaction with usability to provide all stakeholders with up-to-date information on the quality of each product, enabling them to make informed decisions that benefit the supply chain ecosystem. Besides, our experimental analysis shows that, the overall framework using PoA has less gas consumption in terms of transaction and execution costs. Other key issues in the supply chain, including electronic payments and proof of delivery, these will be addressed in future works.

## Supplemental Information

10.7717/peerj-cs.801/supp-1 Supplemental Information 1 Logs of successful seed purchase from SSC.

10.7717/peerj-cs.801/supp-2 Supplemental Information 2 Logs of successful grain product sell to Consumer.

10.7717/peerj-cs.801/supp-3 Supplemental Information 3 Framework codes. The codes for this work is also available in Github public repository on the following link: https://github.com/sysbel07/RiceChain.git

## Additional Information and Declarations

### Data Availability

The following information was supplied regarding data availability: The raw data are available in the Tables. The codes are available at GitHub: https://github.com/sysbel07/RiceChain.git.
