# Supplementary figures and images for: RiceChain: secure and traceable rice supply chain framework using blockchain technology
Source: PeerJ Comput Sci. 2022 Jan 12;8:e801. doi: 10.7717/peerj-cs.801 (PMC8771771; doi:10.7717/peerj-cs.801)

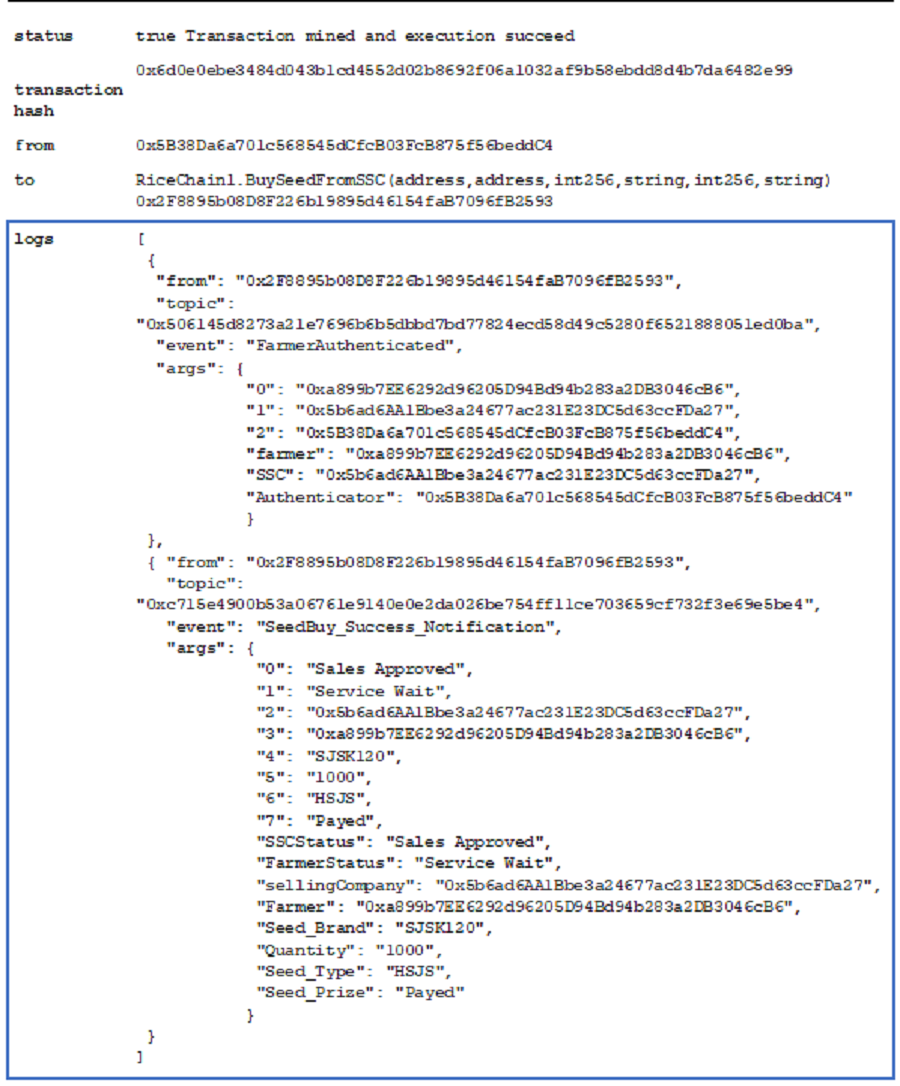

Supplement: Supplemental Information 1 [file peerj-cs-08-801-s001.png]

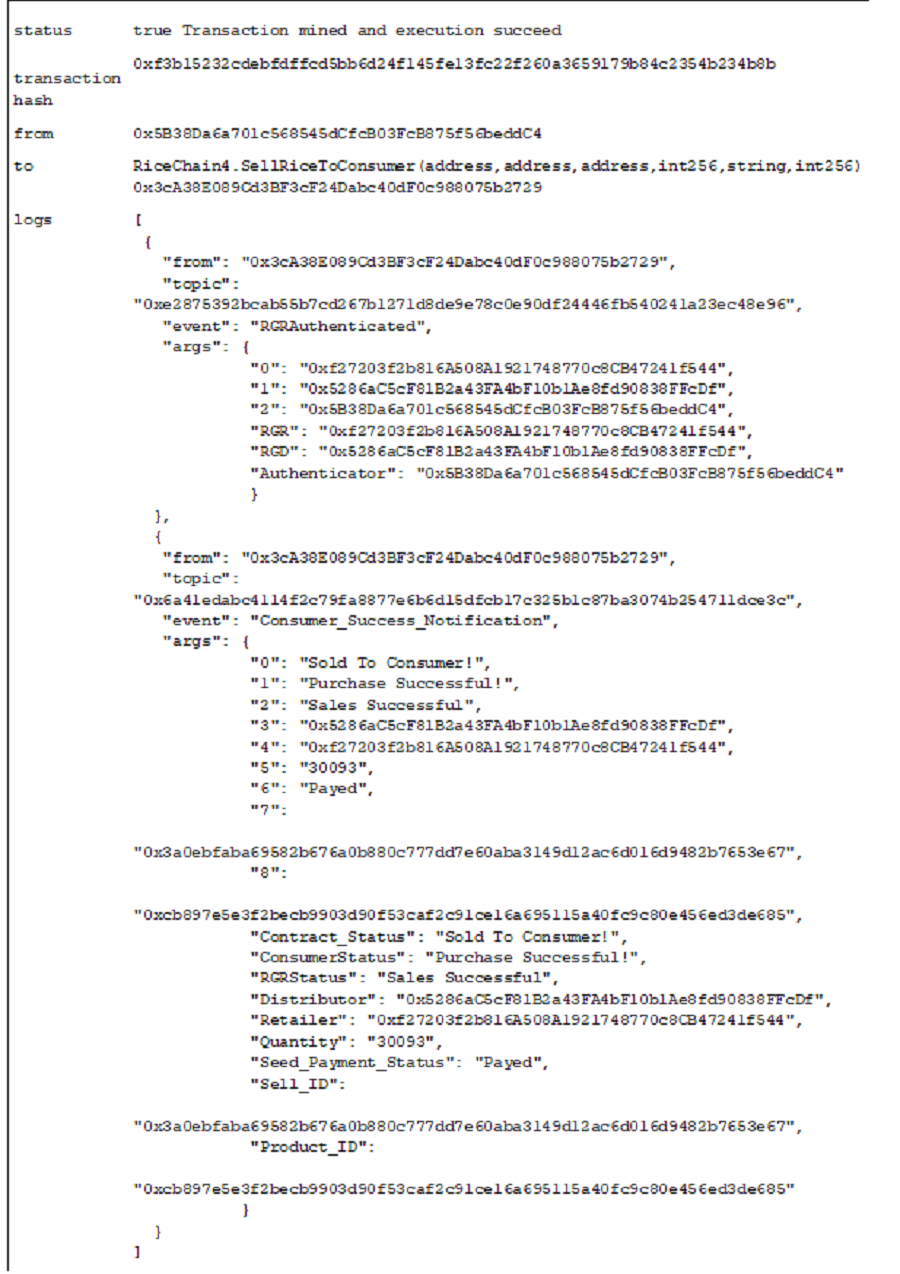

Supplement: Supplemental Information 2 [file peerj-cs-08-801-s002.png]
